# Role of Divalent Cations in HIV-1 Replication and Pathogenicity

**DOI:** 10.3390/v12040471

**Published:** 2020-04-21

**Authors:** Nabab Khan, Xuesong Chen, Jonathan D. Geiger

**Affiliations:** Department of Biomedical Sciences, University of North Dakota School of Medicine and Health Sciences, Grand Forks, ND 58203, USA; nabab.khan@und.edu (N.K.); xuesong.chen@und.edu (X.C.)

**Keywords:** human immunodeficiency virus type-1, transactivator of transcription, HIV-1 associated neurocognitive disorders, divalent cations, endolysosomes

## Abstract

Divalent cations are essential for life and are fundamentally important coordinators of cellular metabolism, cell growth, host-pathogen interactions, and cell death. Specifically, for human immunodeficiency virus type-1 (HIV-1), divalent cations are required for interactions between viral and host factors that govern HIV-1 replication and pathogenicity. Homeostatic regulation of divalent cations’ levels and actions appear to change as HIV-1 infection progresses and as changes occur between HIV-1 and the host. In people living with HIV-1, dietary supplementation with divalent cations may increase HIV-1 replication, whereas cation chelation may suppress HIV-1 replication and decrease disease progression. Here, we review literature on the roles of zinc (Zn^2+^), iron (Fe^2+^), manganese (Mn^2+^), magnesium (Mg^2+^), selenium (Se^2+^), and copper (Cu^2+^) in HIV-1 replication and pathogenicity, as well as evidence that divalent cation levels and actions may be targeted therapeutically in people living with HIV-1.

## 1. Introduction

Divalent cations help regulate vital cellular functions and accumulation of divalent cations has been implicated in healthy aging as well as the pathogenesis of various neurodegenerative diseases and cancer [1,2,3,4]. Underlying such physiological regulatory events and pathological conditions are divalent cation-dependent metalloproteins and metalloenzymes [5,6,7,8,9]. These proteins are required for critical cellular functions, including signal transduction [10], cell division [11], cell excretions [12], gene transcription [13], immune response and regulation [14,15], and cell adhesion [16,17]. Therefore, it is not surprising that multiple receptors and ligands exist in eukaryotic cells that are capable of sorting, transporting, and delivering divalent cations [18,19,20,21,22,23,24,25,26,27,28,29,30].

Additionally, divalent cations play prominent roles in host–pathogen interactions [31,32,33]. And, as above, homeostatic regulation of divalent cation levels is important, because they can affect microbial infection [34,35]. This is certainly true for human immunodeficiency virus type-1 (HIV-1), because levels of divalent cations change during HIV-1 infection. However, it is not yet clear if the changes in divalent cation concentrations construe a defense strategy of the host or the virus [36,37,38,39,40,41]. Thus, it is important to focus additional attention on the involvement of divalent cations in HIV-1 replication and infection.

## 2. HIV-1 Infection and Replication

More than 40 million people are currently living with HIV-1, the causative agent of acquired immunodeficiency syndrome (AIDS). Early in the HIV-1/AIDS pandemic, the high rate of mortality in people living with HIV-1 (PLWH) was due mainly to opportunistic infections. However, with the use of modern anti-retroviral therapeutic (ART) strategies, PLWH are now living almost full life spans [42]. Hence, HIV-1 has become a chronic managed disease.

**Figure 1:**
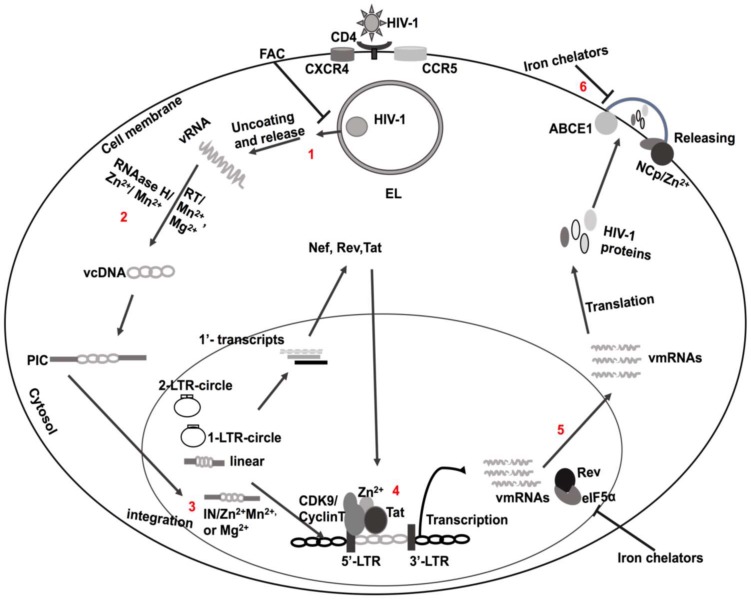
Roles of divalent cations in the HIV-1 life cycle and pathogenicity: **1.** HIV-1 infects cells by first binding gp120 with CD4 receptors and CXCR4/CCR5 co-receptors. Post endocytosis, HIV-1 escapes from endolysosomes (EL) into the cytosol, where it is uncoated. **2.** Viral RNA is reverse-transcribed into viral DNA. During reverse transcription, Mn^2+^, Mg^2+^, and Zn^2+^ control reverse transcription by regulating RNAse H and RT enzymes. Prior to integration, non-integrated DNA transcribes synthesis of early proteins Tat, Rev, and Nef. **3.** IN requires divalent cations, including Mn^2+^/Mg^2+^ and Zn^2+^, for proper integration into the host genome. **4.** Post integration, Tat elongates and terminates the transcription process of HIV-1. Zinc enhances interactions between Tat and the host factors (CycT1 and CDK9) with the HIV-1 LTR promoter for proper transcription. **5**. Post transcription, HIV-1 transcripts (viral mRNAs) are transported to the cytosol by the Rev protein with the help of eIF5α. To be functional, eIF5α needs iron. After translation, HIV-1 is transported to the cell membrane, where it assembles progeny virion particles. **6.** During virus assembly, the virus needs a cellular protein, ABCE1 (ATP-binding cassette sub-family E member 1), for proper assembly by enhancing accessibility of the HIV-1 Gag protein to the virus packaging site. However, the NCp protein is also important for virus assembly to arrange the Gag protein in virion particles. Indeed, zinc is required for Gag dimerization and trafficking to the cell membrane. However, levels of divalent cations are altered in HIV-1 infection and are differentially regulated as HIV-1 disease progresses. Supplementation of divalent cations may either be beneficial or harmful to the virus, and extracellular FAC can inhibit HIV-1 escape from endolysosomes. Abbreviations: CD4: cluster differentiation 4, CXCR4: Cysteine-X-Cysteine chemokine receptor type 4, CCR5: Cysteine-Cysteine chemokine receptor type 5, Gp120: glycoprotein 120, EL: endolysosome, FAC: ferric ammonium citrate, vRNA: viral RNA, RNAse H: ribonuclease H, RT: reverse transcriptase, vcDNA: viral complementary DNA, PIC: pre-integration complex, Nef: Negative regulatory factor, IN: Integrase, CDK9: cyclin-dependent kinase 9, LTR: long terminal repeat, Tat: transactivator of transcription, vmRNAs: viral messenger RNAs, Rev: regulator of expression of virion particles, eIF5α: translation initiation factor 5α, and NCp: nucleocapsid protein.

HIV-1 is a single-stranded RNA lentivirus whose genome is encoded by nine different genes; each gene is transcribed into specific proteins. HIV-1 enters cells by first fusing its viral coat protein gp120 with host receptor proteins, especially CD4 (Figure 1) [43]. Before integration, non-integrated DNA generates all three classes of viral transcripts; the multiply spliced, single spliced, and full-length transcripts. The multiply spliced, early transcripts (*tat, rev* and *nef*) generate early proteins; Tat, Rev, and Nef. These early viral proteins promote virus replication [44]. With the active transcription process, new transcripts are then produced and translated; these include mRNAs for the Gag–Pol polyprotein, and the virion’s genomic RNA (Figure 1) [44,45,46]. During transcription as well as post-transcription, new virus particles are assembled in and released from infected cells to initiate bystander cell infection [47].

HIV-1 Tat is essential for initiating, elongating, and terminating HIV-1 replication, especially early in the infection cycle [48]. The initiation and elongation of transcription is further aided by the ability of HIV-1 Tat to enhance the association of multiple host factors at the HIV-1 LTR promoter site [49,50,51,52]. HIV-1 Tat is a virotoxin that is actively secreted from infected cells [53,54,55] and it continues to be implicated in the pathogenesis of HIV-1-associated neurocognitive disorders (HAND) [56,57,58,59]. Because of its importance as a regulator of HIV-1 replication and the pathogenesis of HAND [60,61], this review will focus mainly on HIV-1 Tat, but other HIV-1 viral factors will be discussed as well.

## 3. Structural and Functional Domains of HIV-1 Tat

Post-infection, HIV-1 Tat is produced from the primary transcript of HIV-1. HIV-1 Tat is composed of 86 to 101 amino acids and six distinct domains have been characterized according to their constituent amino acids and their functionality [62,63,64]. Domain one contains proline-rich acidic amino acids, which is referred to as N-terminal domain (1–21 amino acids). The second domain (21–37 amino acids) has seven cysteine residues (Cys22, Cys25, Cys27, Cys30, Cys31, Cys34, and Cys37), the sites at which disulfide scaffolds are mainly formed under the influence of divalent cations [65,66]. Zn^2+^ appears especially important at these sites, because it facilitates the formation of bridges between Tat and CyclinT1; the result is advanced HIV-1 transcription [67]. Genetic variations in the cysteine-rich domain decrease associations between cellular proteins and transcription factors with the HIV-1 LTR promoter (Figure 1). The third domain (amino acids 38–48) is composed of LGISYG amino acids that form a hydrophobic core region. The fourth domain is a basic arginine-rich motif (49RKKRRQRRR57) [68,69], and this region plays a key role in HIV-1 Tat nuclear localization, HIV-1 Tat binding to the HIV-1 promoter region trans-activation response (TAR) [69,70], and the ability of HIV-1 Tat to penetrate plasma membranes [71,72,73]. The arginine-rich sequence has been used to help deliver a wide variety of macromolecules into cells [74,75]. The fifth domain is a basic glutamine-rich region (residues 58–72) with highly variable genetics. Finally, the sixth domain (amino acids 73–101) is thought to be involved in the advancement of HIV-1 infectivity by supporting transcription [76,77].

## 4. Tat-Mediated Activation of Transcription

HIV-1 transcription starts with RNA polymerase II (RNA II) binding to the long terminal repeat (LTR) promoter along with other transcription factors [78,79,80]. HIV-1 Tat elongates the transcript by delivering essential transcription factors and cofactors to the TAR region of the LTR promoter [80,81,82,83]. Moreover, HIV-1 Tat generates a productive super elongation network by recruiting p-TEFb (cyclin-dependent kinase CDK9 and cyclin T1 complex) and other cellular cofactors to the TAR domain [84,85,86,87,88] and by discharging p-TEFb from the dormant complex of 7SK small nuclear ribonucleoprotein (7SKsnRNP) and hexamethylene bis-acetamide-inducible protein 1 (HEXIM-1) [89,90]. When Tat-TAR interaction problems occur, HIV-1 transcription prematurely terminates and HIV-1 can reside inside cells in the latent phase [91,92]. However, HIV-1 can exhibit latency breakthrough resulting from modulation of Tat and TAR interactions by stimulatory factors generally [93,94] and specifically by divalent cations [95,96].

## 5. Zinc (Zn^2+^)

More than 3000 metalloenzymes and metalloproteins require Zn^2+^ to catalyze processes and affect cellular functions [97,98,99]; examples, include zinc finger proteins, metallothioneins, neuropeptides, hormone receptors, and copper–zinc superoxide dismutase (SOD) [100,101,102,103]. Additionally, Zn^2+^ plays an essential role in immune responses, as well as maturation and differentiation of immune cells [103,104] by sequestering zinc [105,106] and inducing macrophage toxicity [106,107].

Zn^2+^ deficiency has been described for several diseases, including sickle cell anemia, malnutrition, cancer, alcoholism, uremia, and various infections. For PLWH, serum levels of Zn^2+^ decreased with disease progression [108,109,110,111,112,113,114,115,116,117] and clinical symptom similarities exist between HIV-1/AIDS and Zn^2+^ deficiency [113,114,118]. Further, higher mortality rates occurred in PLWH with low levels of Zn^2+^ [119,120,121,122], and Zn^2+^ deficiency and increased levels of tumor necrosis factor-α (TNF-α) were found in people with AIDS [123,124,125]. Zn^2+^ suppresses TNF-α expression by maintaining higher levels of inflammatory cytokine interleukin-4 (IL-4) [126,127]. Thus, adequate levels of zinc might delay disease progression in PLWH [128].

Zn^2+^ is an essential cofactor of the anti-oxidative enzyme Cu-Zn SOD. Because PLWH have lower levels of Zn^2+^, SOD levels are reduced, and this may lead to increased lipid peroxidation, oxidative stress, and ferroptosis [129,130]. In contrast, HIV-1 infection was restricted when cells were supplemented with Cu-Zn SOD [131]. However, HIV-1 also uses zinc for replication and progression of infection; Zn^2+^ is a cofactor for integrase (IN), nucleocapsid (NCp), HIV-1 Tat, and viral infectivity factor (Vif).

Integrase is an HIV-1 encoded enzyme that catalyzes integration of the HIV-1 DNA into the host genome and Zn^2+^ enhances IN activity by promoting its multimerization; it does so by binding to IN cysteine domains [132,133,134,135]. The site of action of Zn^2+^ is likely the HIV-1 NCp protein, a nucleic acid-binding protein composed of one or two cysteine-rich domains (CysteineX_2_CysteineX_4_HistidineX_4_Cysteine: CCHC motif) flanked by basic amino acids [136,137,138]. Moreover, NCp also regulates the trafficking of HIV-1 polyprotein Gag [139], HIV-1 genomic RNA (gRNA) dimerization [140,141], and generation of infectious progeny virion particles (Figure 1) [142,143,144].

HIV-1 Tat contains zinc-binding cysteine-rich domains, and once bound, Tat dimerizes [62,63,65,145,146,147,148,149,150]. Zn^2+^-mediated bridges with Cys261 domains of CycT1 facilitate Tat interactions with the HIV-1 LTR promoter region TAR [67]; these findings were confirmed using mutagenesis approaches (Figure 1) [60,67]. Following structural modification by Zn^2+^, Tat interacts with other cellular partners, including Cyclin T1 [67], N-methyl-d-aspartate (NMDA) receptors [151,152], and microtubules [150]. Thus, Zn^2+^ may affect Tat-induced HIV-1 transcription through various cellular processes [153].

Vif is an HIV-1 accessory protein that increases HIV-1 infection by inducing proteasomal degradation of anti-viral factor APOBEC3G [154]. APOBEC3G catalyzes deamination of deoxycytosine to deoxyuracil and thereby inhibits HIV-1 infectivity [155]. Vif has a cysteine repeat domain that binds Zn^2+^ and causes structural modification of Vif from an alpha- to a beta-sheet structure [156]. The folded beta-sheet structure of Vif promotes selective assembly of the Cullin5-E3 ligase and selects APOBEC3G for degradation by the proteasomal pathway [154]. The net result of increasing the degradation of anti-viral factor Vif is enhanced HIV-1 infection.

Zinc is also important for anti-viral activity of the zinc finger protein (ZAP), a host factor for multiple viruses [157]. ZAP recruits the 5′- and 3′-mRNA degradation machinery, the net result being decreased HIV-1 mRNAs levels [158]. The tripartite motif 25 protein (TRIM25) is an essential factor for antiviral activity of the ZAP and decreases in protein expression levels of TRIM25 suppress antiviral activity of the ZAP [159].

## 6. Ferrous Iron (Fe^2+^)

Iron controls various cellular functions, including lysosomal activity, mitochondrial respiration, DNA synthesis, blood formation, oxygen consumption and transportation of oxygen, hormone synthesis, and cellular signaling [160]. Many metalloproteins and metalloenzymes require iron as a cofactor and are dependent on it for their actions [161]. Multiple iron-binding proteins, including lactoferrin, alpha-lipoic acid, calprotectin, transferrin, ferritin, heme oxygenase-1, ferroportin, myoglobin, mitoferrin, and hepcidin [162,163,164,165], help maintain iron homeostasis.

Homeostatic regulation of iron levels starts with the uptake of iron by intestinal enterocytes through divalent metal transporters (DMT1) [166]. Ferric iron binds with transferrin in blood [167] and iron-bound transferrin is endocytosed into acidic endolysosomes [167,168,169,170]. Once endocytosed, iron is reduced to ferrous iron, a process catalyzed by the six-transmembrane epithelial antigen of prostate 3 (STEAP3) [171]. Ferrous iron can then be transported into the cytosol through endolysosome-resident channels, including DMT1 [172] and mucolipin-1 (TRPML1) [173]. Cytosolic iron can be up-taken into other organelles through various cation channels, or it can be exported extracellularly by ferroportin.

Iron is fundamental to the production of reactive oxygen species (ROS); hydroxyl radicals are formed by the Fenton reaction [174]. When excessive, ROS causes mitochondrial dysfunction, DNA destruction [175], lipid peroxidation [175,176], and the iron-based cell death process ferroptosis [177,178]. ROS levels can be modulated by a number of factors, including SOD, catalase, glutathione peroxidase, glutathione, cysteine, ascorbic acid, and alpha-tocopherol (vitamin E) [179,180].

Iron also plays essential roles in multiple stages of HIV-1 infection, including translation of viral mRNAs, virus packaging, reverse transcription, HIV-1 transcription, and nuclear factor kappa-light-chain enhancer of activated B-cells (NF-kB) activation [95,181,182]. With HIV-1 progression, iron accumulates in muscle, brain white matter, myocytes, and macrophages [183,184,185]; the findings were confirmed in patients with thalassemia, with haptoglobin 2-2 polymorphism, and those taking iron supplements [185]. Moreover, increased HIV-1 load correlates with increased ferritin levels in the serum of non-anemic HIV-1 infected women [186]. Additionally, higher mortality rates and increased iron levels were observed in PLWH [187].

HIV-1 proteins can disturb iron homeostasis as well as enhance HIV-1 replication and disease progression. The HIV-1 accessory protein negative regulatory factor (Nef) enhances intracellular levels of iron through the actions mediated by the human homeostatic iron regulator protein (HFE) [185]. Iron overload also occurs with HFE mutation and with hemochromatosis. Nef-induced mis-trafficking of the HFE protein to perinuclear regions near the trans-Golgi network might lead to enhanced HIV-1 infection by increasing levels of intracellular iron.

Iron regulatory host proteins are also involved in the completion of the HIV-1 life cycle. The ATP-binding cassette subfamily E member 1 (ABCE1) protein is an iron–sulfur-containing metalloprotein [188] that is involved in the assembly of newly synthesized virions at the cell membrane by direct interaction with the HIV-1 Gag protein (Figure 1.6) [189]. Furthermore, HIV-1 protein Rev requires host factor eIF5α for transportation of un-spliced HIV-1 mRNAs to the cytosol from the nucleus [190,191], and iron-containing enzyme deoxyhypusine hydroxylase is required to produce hypusine, a vital part of the eIF5α protein (Figure 1.5) [192]. Thus, iron chelators may restrict HIV-1 infection by decreasing the actions of the eIF5α and ABCE1 proteins (Figure 1) [193].

Macrophages play an important role in HIV-1 infection, pathogenesis, and latency [194]. They also play important roles in regulating iron levels in red blood cells (RBCs) [195,196]. During hemolysis, heme stimulates iron regulatory host protein transcription of ferroportin and HO-1 [196]. The increased levels of ferroportin results in decreased levels of intracellular iron in stimulated macrophages by exporting more iron from cells [196,197]; mutated inactive ferroportin increases levels of intracellular iron [198]. Hepcidin is another iron regulatory host protein that enhances levels of intracellular iron, because it degrades ferroportin in endolysosomes [163]. The above findings are consistent with the findings that HIV-1 transcription is increased with high levels of intracellular iron by hepcidin-mediated decreases in ferroportin [199].

In sickle cell disease (SCD), the hepcidin protein expression levels are low [200,201] and the progression of HIV-1 infection is delayed [202,203]. SCD is a genetic disease with a single mutation in the beta-globin gene that is characterized by chronic hemolytic anemia (hemolysis) [204]. With hemolysis, there is an increased release of heme from hemoglobin and increased expression levels of multiple iron regulatory factors, including ferroportin, HO-1, p21, and biliverdin reductase. However, somewhat paradoxically, intracellular iron levels are decreased [205] and, because of this, activity levels of CDK2 are decreased [206,207]. Decreased CDK2 activates SAMHD1 (sterile alpha motif and histidine/aspartic acid (HD) domain-containing protein 1) by enhancing its dephosphorylation [208]. HIV-1 reverse transcription is restricted by dephosphorylated SAMHD1 (active). However, at adequate levels of iron, active CDK2 increased inactive levels of SAMHD1 and increased HIV-1 replication [209,210]. Elevated levels of the p21 protein are linked to increased activation of SAMHD1 in HIV-1 elite controllers [211], increased Egr-1 (early growth response) [212], which is regulated by the hypoxia-inducible factor (HIF-1a) [212,213], and SCD when iron levels are low [214]. Furthermore, various studies have shown that hemin-produced HO-1 efficiently restricts HIV-1 infection by reducing intracellular iron levels [214,215]. HIV-1 infection was restricted by hemin treatment; the findings were similar to those showing inhibition of HIV-1 in individuals with SCD [216,217].

Protein phosphatase-1 (PP1) activity increases HIV-1 transcription, likely because of its effects on iron and HIF-1α. A low level of iron decreases PP1 catalytic activity and increases HIF-1α [218,219]. PP1 also increases HIV-1 transcription by releasing CDK9 from the inactive complex of 7SKRNP and HEXIM1 [220,221]. PP1 expression can be negatively regulated by hypoxia either by limiting levels of mRNA [222] or mRNA transports to the nucleus [223,224]. Hypoxia suppressed HIV-1 replication by decreasing CDK9 activity and/or inactivating PP1 [224,225]. Nonetheless, HIV-1 can overcome the effects of the PP1 protein on HIV-1 replication by enhancing PP1 transport to the nucleus by Tat and increasing the actions of CDK9.

Because iron is important for HIV-1 replication, intracellular iron chelators have been investigated for their ability to control HIV-1 (Figure 1) [226,227]. Deferiprone, fungicide, and ciclopirox all reduced levels of HIV-1 gene expression at the transcription level by targeting cellular factor eIF5α [193,206,207]. Additionally, deferasirox, Bp4aT, Bp4eT, and iron chelator 311 suppressed HIV-1 replication by targeting CDK2 and CDK9 [228,229,230]. Moreover, CDK2 inactivation by iron chelators was associated with cyclin-dependent kinase inhibitors p27 (Kip1) and p21 (CIP1/WAF1) [229,230]. Higher levels of p21 have been reported in PLWH [231]. However, HIV-1 production was increased when p21 expression levels were decreased [211]. Thus, p21 may restrict HIV-1 infection through anti-CDK2/9 properties [231,232,233] and CDK2 phosphorylation effects of HIV-1 Tat may enhance HIV-1 infection [234,235,236]. In addition, cyclin A (S phase transition) and cyclin E (G1/S phase transition) can regulate CDK2 at different cell cycle stages [236].

Extracellular iron can also restrict HIV-1 replication and infection. Extracellular ferric ammonium citrate (FAC) restricted HIV-1 infection by inhibiting the release of HIV-1 from endolysosomes; it increased the fusion of vesicles in host cells (Figure 1) [237]. Iron may also decrease HIV-1 by restricting the ability of HIV-1 Tat to bind to the TAR region of the HIV-1 LTR promoter [238]. Iron overload also suppressed HIV-1 replication by decreasing Rev cofactor eIF5α [239].

Iron can also play an important role in HIV-1 latency [240]. Iron can reactivate HIV-1 replication by inducing oxidative stress [241]. On the other hand, iron chelators may restrict HIV-1 reactivation by decreasing oxidative stress, reducing expression of anti-HIV-1 factor beta-catenin, and blocking cell proliferation [242,243]. However, much more work is needed to understand better the possible therapeutic use of iron chelators in HIV-1 latency, including reservoirs in the CNS [58].

## 7. Manganese (Mn^2+^) and Magnesium (Mg^2+^)

Manganese is a cofactor of numerous metalloenzymes, including manganese superoxide dismutase (Mn-SOD) [244,245], pyruvate carboxylase [246], and glutamate synthetase [247,248]. Manganese is also required for mucopolysaccharide metabolism, oxidative phosphorylation, and oxidative stress [249]. Increased oxidative stress can result in higher levels of HIV-1 and increases in Mn-SOD have been observed in HIV-1 infection; increased Mn-SOD may help protect HIV-1-infected cells from cell death [250,251].

The ability of manganese to control HIV-1 replication appears to be mediated through its actions on reverse transcriptase (RT) and integrase (IN) enzyme activity. RT is essential for reverse transcription of viral DNA from viral RNA. RT is composed of the p66 and p51 subunits driven by viral proteases from the Gag-Pol polyprotein and RT activity requires divalent cations, including Mn^2+^ and Mg^2+^ [252]. The two subunits are necessary for DNA polymerase and RNAase H activity and produce double-stranded DNA (ds-DNA) [253,254]. RNAase H (p51) has binding sites for divalent cations [255,256,257]; Mn^2+^ and Mg^2+^ binding at D442, E478, D498, and D549 results in stimulation of enzymatic activity (Figure 1) [258,259]. Moreover, Mn^2+^ can modify RT substrate specificity and increase RT mutations [260,261].

IN is essential for the integration of HIV-1 DNA into the host genome [262,263,264] and Mn^2+^ and Mg^2+^ are known cofactors for integrase activity [265]. IN contains three domains; N-terminal, C-terminal, and catalytic domains [265]. The N-terminal domain contains a highly conserved cysteine repeat domain (the CCHC domain), which is a binding site of Zn^2+^ ions; the binding of Zn^2+^ stabilizes IN and induces IN multimerization [132]. The active site contains an extremely conserved region that is required for Mn^2+^ and Mg^2+^ binding as well as viral integration (Figure 1.3) [135,266] into the viral but not the host DNA [264]. Some IN inhibitors restrict the integration process by chelating Mn^2+^ or Mg^2+^ cations, for example, catechols and beta-ketoenols [267]. Therefore, the IN enzyme is a unique and favorable therapeutic target to inhibit HIV-1 infection.

## 8. Selenium (Se^2+^)

Selenium is essential for cellular antioxidant defense mechanisms, including glutathione peroxidase, SOD, and other selenoproteins [268,269]. Inadequate levels of selenium are associated with gastroenteritis and dermatitis, impaired thyroid hormone metabolism [270], cancers [271], male subfertility, liver dysfunctions [272], weakened immune functions [273], liver dysfunctions, mood disorders, and progression of HIV-1 infection and mortality [38,121,122,274]. With lower levels of selenium, PLWH have higher levels of oxidative stress, increased opportunistic infections, increased viral load, and higher mortality rates [122,274,275,276].

Therapeutically, it is recommended that people ingest selenium (200 μg/day) and studies have shown that this reduces viral load, diminishes HIV-1 infection of monocytes, and reduces the number of CD4+ T-cells [277,278,279,280,281,282]. However, not all studies found protective effects of selenium against the HIV-1 viral load and CD4+ cells in PLWH [283,284]. Selenium supplementation has also been shown to be protective against strokes, possibly by suppressing oxidative stress and blocking ferroptosis [285].

## 9. Copper (Cu^2+^)

Copper, too, is an essential cofactor of multiple metalloenzymes and metalloproteins as well as an important part of the cellular anti-oxidative system [286]. Copper has anti-microbial properties by radical and non-radical mechanisms [287] and by phagosome-burst and -maturation mechanisms [107,288]. However, pathogens can resist the actions of copper by sorting it into siderophore structures [289].

Copper, zinc, cysteine, and glutathione all effectively inhibit HIV-1 [119,120,290,291]. Cysteine and glutathione are essential parts of SOD and catalase, which block HIV-1-mediated oxidative stress and its consequences [292,293,294,295]. Copper and zinc are released from metallothionine by glutathione and thereby become biologically active. Zn^2+^ can either facilitate HIV-1 infection or it can inhibit the production of mature and infectious virus particles by inhibiting HIV-1 protease activity [296]. Similar to zinc, copper can inhibit HIV-1 protease activity by binding directly to cysteine amino acids [297]. Therefore, sufficient Cu^2+^, Zn^2+^, glutathione, and cysteine can control HIV-1 infection [290]. Moreover, extracellular copper/zinc SOD has anti-HIV-1 effects [298] and reduces neurotoxic effects of HIV-1 proteins [299,300] by controlling oxidative stress. Additionally, copper oxide contained in latex, polymeric materials, filter matrices and fibers has virucidal activity [301]; copper-coated filters neutralized HIV-1 virus particles [302,303] and copper oxide restricted HIV-1 transmission from breast milk [304].

## 10. Roles of Divalent Cations in HIV-1 Tat-Mediated Pathogenicity

**Figure 2:**
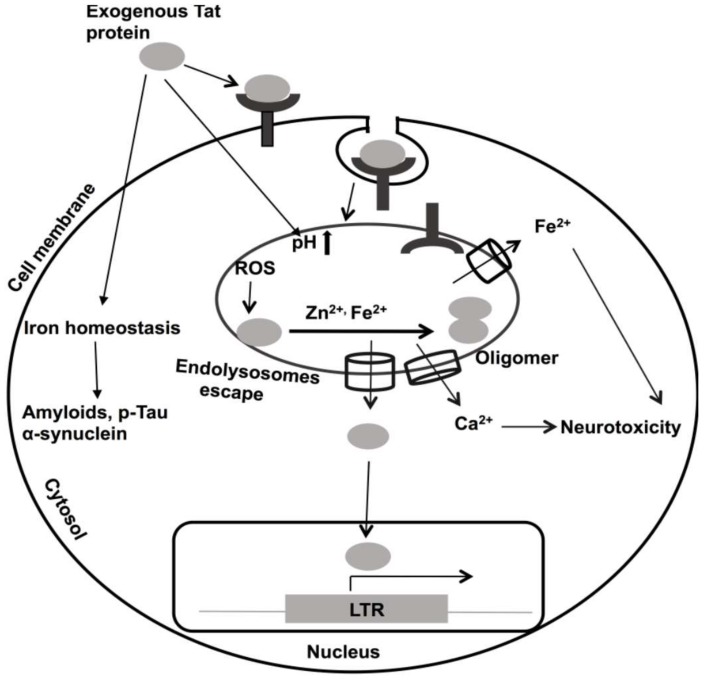
Roles of divalent cations in HIV-1 Tat-mediated pathogenicity. Extracellular Tat is a neurotoxin implicated in the pathogenesis of HIV-1-associated neurocognitive disorders (HAND). Tat enters bystander cells by receptor-mediated endocytosis. Internalized Tat disturbs endolysosome functions, including de-acidification. When de-acidified, Tat is released from endolysosomes and enters the nucleus where it activates HIV-1 LTR transactivation. Endolysosomes contain divalent cations and these can induce Tat oligomerization. Because Tat oxidizes rapidly, Tat can be oxidized by iron-induced reactive oxygen species (ROS). Disturbed iron homeostasis may be involved in increasing levels of various neurotoxic substances, including β-amyloid, p-Tau, and α-synuclein.

HIV-1 Tat continues to be implicated in the pathogenesis of HAND [305,306,307]. As such, it is known as a virotoxin [306,307]. In PLWH, HIV-1 Tat is present in plasma and cerebrospinal fluid (CSF), and its levels can stay elevated even when virus levels were effectively controlled by ART [308]. HIV-1 negatively affects neurons by increasing levels of intracellular calcium [309,310], increasing ROS [130], and causing bioenergetic failure [311]. HIV-1 Tat contains an arginine-rich domain (Tat 48–60) that causes it to be up-taken into cells by receptor-mediated endocytosis [71,73]; this same feature is used experimentally to enhance the uptake into cells of a wide variety of macromolecules [75]. The receptors to which HIV-1 Tat binds include low-density lipoprotein receptor 1 [55], CXCR4, heparin sulfate proteoglycan [312], and CD26 [56,313,314]. Following its endocytosis, HIV-1 Tat associates with endolysosomes [53,55,315]. However, HIV-1 Tat must escape from endolysosomes to activate the HIV-1 LTR in the nucleus [56,316,317], a process known to be mediated by endolysosome de-acidification and calcium [315,316,317]. However, the underlying mechanisms responsible for Tat escape from endolysosomes remain poorly defined (Figure 2).

In addition to its active secretion from infected cells, HIV-1 Tat and Tat mRNAs can also exit cells via released extracellular vesicles (exosomes) and this released material might then be taken up by an uninfected bystander cell to enhance HIV-1 pathogenicity [318,319]. Secreted Tat has been shown to modify activator protein-1 (AP-1), nuclear factor kappa-light-chain enhancer of activated B-cells (NF-kB), and cAMP responsive element-binding protein (CREB) transcription factors and affect diverse cellular signaling pathways [320,321,322,323,324,325]. Secreted Tat impairs endolysosome membrane integrity and degradation pathways [326]; the changes noted include changes in their pH, distribution patterns, sizes, and membrane integrity [326,327]. Endolysosome de-acidification may also affect the cation contents of endolysosomes, including the cations discussed above: Fe^2+^, Cu^2+^, Mn^2+^, Mg^2+^, Zn^2+^, and Cd^2+^. To varying extents, divalent cations can promote the oligomerization of Tat by binding to its cysteine-rich domain [65,66,328]. As discussed above, Zn^2+^ plays a role in inducing conformational changes to and physiological actions of Tat [149,150,151,152]. In addition to Zn^2+^, iron also induces HIV-1 Tat oligomerization (unpublished) (Figure 2), an action possibly mediated by iron-induced ROS production and iron-induced oxidation and oligomerization of HIV-1 Tat (Figure 2) [329]. These effects of iron may be especially relevant in older PLWH, because iron is aggregated in aged brains, and it is known to induce accumulation of the β-amyloid, p-Tau and α-synuclein proteins [330,331,332].

## 11. Summary

Divalent cations are involved in the pathogenesis of HIV-1 as well as the ability of the host to control HIV-1 replication. However, the extent to which divalent cations are beneficial or harmful to PLWH is not clear. Thus, caution might be advised about divalent cation supplementation to PLWH. This might be especially true for iron; because it is highly abundant, it plays important roles in regulating HIV-1 infection, and iron level are elevated as HIV-1 infection progresses. Further, the use of iron chelators might inhibit HIV-1 replication and progression. Moreover, the iron homeostasis disturbed by HIV-1 and HIV-1 Tat may regulate expression of anti-HIV-1 cellular factors and immune responses by iron regulatory proteins hepcidin and ferroportin [333]. More comprehensive examinations are required to determine biological effects of divalent cations in HIV-1 infection and this information might inform the development of novel therapeutic strategies.
